# Cryo-EM structure of infectious bronchitis coronavirus spike protein reveals structural and functional evolution of coronavirus spike proteins

**DOI:** 10.1371/journal.ppat.1007009

**Published:** 2018-04-23

**Authors:** Jian Shang, Yuan Zheng, Yang Yang, Chang Liu, Qibin Geng, Chuming Luo, Wei Zhang, Fang Li

**Affiliations:** 1 Department of Veterinary and Biomedical Sciences, College of Veterinary Medicine, University of Minnesota, Saint Paul, MN, United States of America; 2 Department of Diagnostic and Biological Sciences, School of Dentistry, University of Minnesota, Minneapolis, MN, United States of America; 3 Characterization Facility, College of Science and Engineering, University of Minnesota, Minneapolis, MN, United States of America; Loyola University Chicago Stritch School of Medicine, UNITED STATES

## Abstract

As cell-invading molecular machinery, coronavirus spike proteins pose an evolutionary conundrum due to their high divergence. In this study, we determined the cryo-EM structure of avian infectious bronchitis coronavirus (IBV) spike protein from the γ-genus. The trimeric IBV spike ectodomain contains three receptor-binding S1 heads and a trimeric membrane-fusion S2 stalk. While IBV S2 is structurally similar to those from the other genera, IBV S1 possesses structural features that are unique to different other genera, thereby bridging these diverse spikes into an evolutionary spectrum. Specifically, among different genera, the two domains of S1, the N-terminal domain (S1-NTD) and C-terminal domain (S1-CTD), diverge from simpler tertiary structures and quaternary packing to more complex ones, leading to different functions of the spikes in receptor usage and membrane fusion. Based on the above structural and functional comparisons, we propose that the evolutionary spectrum of coronavirus spikes follows the order of α-, δ-, γ-, and β-genus. This study has provided insight into the evolutionary relationships among coronavirus spikes and deepened our understanding of their structural and functional diversity.

## Author summary

Because of their structural and functional diversity, coronavirus spike proteins represent a model system for studying viral evolution. Their evolutionary relationships and history pose major puzzles in virology. A critical missing piece in understanding the evolution of coronavirus spikes is the unavailability of the tertiary structure of γ-coronavirus spikes. Here we determined the first cryo-EM structure of avian infectious bronchitis coronavirus (IBV) spike from the γ-genus. The IBV spike contains structural features that are unique to different other genera; hence it brings the spikes from different coronavirus genera into one evolutionary spectrum, with itself sitting in the middle of this spectrum. The order of the evolutionary spectrum is α-, δ-, γ-, and β-genus. Importantly, through comparisons between IBV spike and other coronavirus spikes, this study illustrates how coronavirus spikes have achieved structural and functional diversity through evolution to guide viral entry into host cells.

## Introduction

As large enveloped RNA viruses, coronaviruses are capable of adapting to new hosts with relative ease through mutations and recombinations [1–3]. As a result, coronaviruses infect a wide range of mammalian and avian species, and have genetically evolved into four major genera: α, β, γ, and δ [4]. Coronaviruses from the four genera all contain envelope-anchored spike proteins that mediate viral entry into host cells [5, 6]. During viral entry, the spikes bind to host receptors through their S1 subunits and then fuse viral and host membranes through their S2 subunits. On the one hand, the spikes interact with host receptors and other host factors, hence needing to evolve for better adaptation to these host factors [7–10]. On the other hand, they are exposed to the host immune system, thereby needing to evolve to evade the host immune surveillance [11–14]. Consequently, the spikes are the most divergent among all coronavirus proteins [6]. The S1 subunits are particularly divergent, with little or low sequence similarities across different genera [15]. How coronavirus spikes have evolved to their current diverse structures imposes a major evolutionary conundrum.

Traces of protein evolution can often be found more reliably in their tertiary structures and related functions than in their primary structures, because proteins generally need to evolve within certain structural and functional constraints [16, 17]. To decipher the evolutionary puzzles surrounding coronavirus spikes, extensive structural studies have been carried out using both X-ray crystallography and cryo-electron microscopy (cryo-EM) [12–14, 18–28]. These studies have resulted in structure determinations of S1 from the α- and β-genera and spike ectodomains from the α-, β-, and δ-genera. Coronavirus spikes exist in two distinct conformations: the pre-fusion structures are present on mature virions and have a clove-like shape with three S1 heads sitting on top of a trimeric S2 stalk [12–14, 18–20]; the post-fusion structures are the membrane-fusion state and have a dumbbell-like shape with three S2 subunits forming a six-helix bundle structure [29–35]. Whereas the structures of S2 from different genera are similar to each other in both the pre- and post-fusion states, the S1 subunits from different genera diverge structurally and they also recognize a variety of host receptors [6, 7]. S1 contains two domains, N-terminal domain (S1-NTD) and C-terminal domain (S1-CTD), either or both of which can function as the receptor-binding domain. The S1 domains from different genera contain structural features that are unique to their genus. S1-CTDs are particularly diverse, with low or no structural similarity across different genera [15]. Overall, these previous studies have provided structural snapshots of coronavirus spikes from α-, β-, and δ-genera. However, because the structures of γ-coronavirus spikes were still missing, we lacked a clear picture of the evolutionary relationships among coronavirus spikes from different genera.

In the current study, we determined the cryo-EM structure of avian infectious bronchitis coronavirus (IBV) spike, the first such structure from the γ genus. The IBV spike possesses structural features that are unique to different other genera, suggesting that it falls in the middle of an evolutionary spectrum of coronavirus spikes. We also discuss how the structural evolution of coronavirus spikes has affected their functions as cell-invading molecular machinery. Overall, our study has filled in a critical gap in the structural, functional and evolutionary studies of coronavirus spikes, and deepened our understanding of viral evolutions in general.

## Materials and methods

### Expression and purification of IBV spike ectodomain

IBV spike gene (virus strain M41; GenBank number ABI26423.1) was synthesized (Genscript) with codons optimized for insect cell expression. Its ectodomain (residues 20–1084) was cloned into pFastBac vector (Life Technologies Inc.) with a N-terminal honeybee melittin signal peptide and C-terminal GCN4 and His_6_ tags. It was expressed in Sf9 insect cells using the Bac-to-Bac system (Life Technologies Inc.) and purified as previously described [21]. Briefly, the protein was harvested from cell culture medium, and purified sequentially on Ni-NTA column and Superdex200 gel filtration column (GE Healthcare). IBV S1-CTD (residues 248–495) was expressed and purified in the same way as the IBV spike ectodomain, although it only contains a C-terminal His_6_ tag and does not contain the GCN4 tag.

### Cryo-electron microscopy

For sample preparation, aliquots of IBV spike ectodomain (3 μl, 0.35 mg/ml, in buffer containing 2 mM Tris pH7.2 and 20 mM NaCl) were applied to glow-discharged CF-2/1-4C C-flat grids (Protochips). The grids were then plunge-frozen in liquid ethane using a FEI MarkIII Vitrobot system (FEI Company).

For data collection, images were recorded using a Gatan K2 Summit direct electron detector in the direct electron counting mode (Gatan), attached to a FEI Titan-Krios TEM, at Arizona State University. The automated software SerialEM [36] was used to collect ~2,000 total movies at 37,700x magnification and at a defocus range between 1 and 3 μm. Each movie had a total accumulated exposure of 53.66 e/Å^2^ fractionated in 50 frames of 200 ms exposure. Data collection statistics are summarized in S1 Table.

For data processing, whole frames in each movie were corrected for beam-induced motion and dose compensation using MotionCor2 [37] and ~1,400 best images were manually selected (we manually discarded micrographs with only carbon field of view or thick ice after motion correction as well as micrographs with defocus parameter higher than 4.5 μm after CTF estimation). The final image was bin-averaged to lead to a pixel size of 1.02 Å. The parameters of the microscope contrast transfer function were estimated for each micrograph using GCTF [38]. Particles were automatically picked and extracted using RELION [39] with a box size of 320 pixels. Initially, ~802,000 particles were subjected to 2D alignment and clustering using RELION, and the best classes were selected for an additional 2D alignment. ~5,000 best particles were applied for creating the initial 3D model using RELION. ~170,000 particles selected from 2D alignment were then subjected to 3D classification and the best class with ~100,000 particles were subjected to 3D refinement to generate the final density map. The final density map was sharpened with modulation transfer function of K2 operated at 300kV using RELION post-processing. Reported resolutions were based on the gold-standard Fourier shell correlation (FSC) = 0.143 criterion, and Fourier shell correction curves were corrected for the effects of soft masking by high-resolution noise substitution [40]. Data processing statistics are summarized in S1 Table.

### Model building and refinement

The initial model of IBV spike ectodomain was obtained by fitting the seven parts (S1-NTD, S1-CTD, two parts of SD1, two parts of SD2, and S2) of the porcine delta coronavirus spike structure (PDB ID: 6B7N) individually into the cryo-EM density map of IBV spike using UCSF Chimera [41] and Coot [42]. Manual model rebuilding was carried out using Coot based on the well-defined continuous density of the main chain; the side chain assignments were guided by the densities of N-linked glycans and bulky amino acid residues. The structural model of the IBV spike in the pre-fusion state was refined using Phenix [43] with geometry restrains and three-fold noncrystallographic symmetry constraints. Refinement and model rebuilding in Coot were carried out iteratively until there were no further improvements in geometry parameters and model-map correlation coefficient. The quality of the final model was analyzed with MolProbity [44] and EMRinger [45]. The validation statistics of the structural models are summarized in S1 Table.

### IBV pseudovirus entry assay

IBV pseudovirus entry assay was carried out as previously described [46]. Briefly, full-length IBV spike gene was inserted into pcDNA3.1 (+) plasmid. Retroviruses pseudotyped with IBV spike and expressing a luciferase reporter gene were prepared through co-transfecting HEK293T cells (source: American Type Culture Collection) with a plasmid carrying Env-defective, luciferase-expressing HIV-1 genome (pNL4-3.luc.RE) and the plasmid encoding IBV spike. The produced IBV pseudoviruses were harvested 72 hours post transfection, and then used to enter DF-1 cells (source: American Type Culture Collection) and HEK293T cells. After incubation for 5 hours at 37°C, the medium was changed and cells were incubated for an additional 60 hours. Cells were then washed with PBS and lysed. Aliquots of cell lysates were transferred to Optiplate-96 (PerkinElmer), followed by addition of luciferase substrate. Relative light units (RLUs) were measured using EnSpire plate reader (PerkinElmer). All the measurements were carried out in quadruplicates.

### Flow cytometry cell-binding assay

Recombinant IBV S1-CTD was assayed for its cell-binding capability using flow cytometry as previously described [13]. Briefly, HEK293T and DF-1 cells were incubated with recombinant IBV S1-CTD containing a C-terminal His_6_ tag (40 μg/ml) at room temperature for 30 minutes, followed by incubation with phycoerythrin (PE)-labeled anti-His_6_ antibody for 30 minutes. The cells were then analyzed for the binding of IBV S1-CTD using flow cytometry.

### Calculation of buried surface area of coronavirus S1-CTDs

The total surface area and buried surface area of coronavirus S1-CTDs were calculated using the PISA server at the European Bioinformatics Institute (http://www.ebi.ac.uk/pdbe/prot_int/pistart.html) [47]. Specifically, for each trimeric spike protein, a PDB file containing all of the six S1 domains (including three copies of S1-CTDs and three copies of S1-NTDs) was submitted to the PISA server, and the total surface area and buried surface area for each S1-CTD were calculated. For the spike proteins used for the above analysis, all their S1-CTDs were in the “lysing down” state. The structures of MERS-CoV and HKU1 spikes were not included in the above analysis because the former contain at least one S1-CTD in the “standing up” state and the latter contains long stretches of missing residues in its S1 domains, both of which would interfere with the above analysis.

## Results

### Overall structure of IBV spike

**Fig 1:**
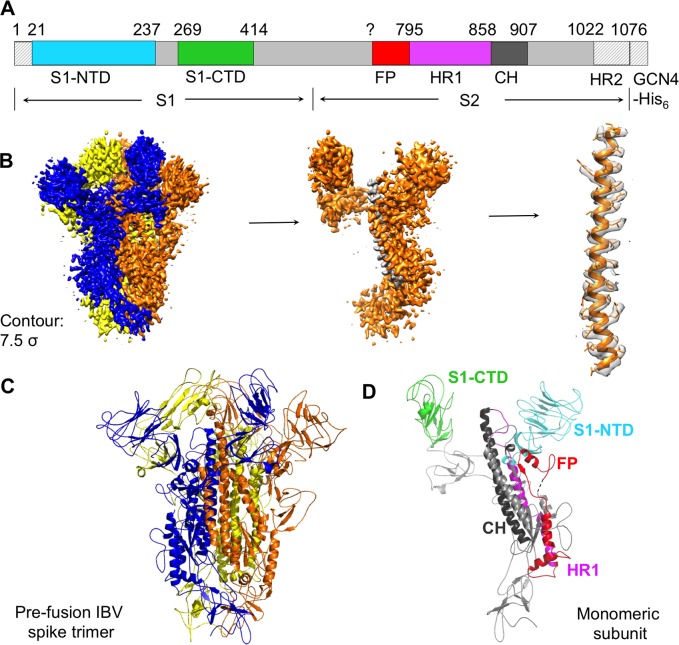
Overall structure of IBV spike ectodomain in the pre-fusion conformation. (A) Schematic drawing of IBV spike ectodomain. S1: receptor-binding subunit. S2: membrane-fusion subunit. GCN4-His_6_: GCN4 trimerization tag followed by His_6_ tag. S1-NTD: N-terminal domain of S1. S1-CTD: C-terminal domain of S1. CH: central helix. FP: fusion peptide. HR1 and HR2: heptad repeats 1 and 2. Residues in shaded regions (N-terminus, HR2, GCN4 tag, and His_6_ tag) were not included in the structural model. Question mark indicates that the exact location of FP is uncertain; the range of FP used for making figures is consistent with a previous structural study on β-genus mouse hepatitis coronavirus spike [18]. (B) Cryo-EM density maps of IBV spike ectodomain with atomic model fitted in. The maps have a contour of 7.5 σ. (C) Cryo-EM structure of IBV spike ectodomain in the pre-fusion conformation. Each of the monomeric subunits is colored differently. (D) Structure of a monomeric subunit in the pre-fusion conformation. The structural elements are colored in the same way as in panel (A). Dotted line indicates residues 702–710 that are missing in the structural model. All structures are viewed from the side.

We constructed the IBV spike ectodomain (from IBV strain M41) in the pre-fusion state by replacing its transmembrane anchor and intracellular domain with a C-terminal GCN4 trimerization tag, followed by a His_6_ tag (Fig 1A). We expressed the protein in insect cells and purified the protein to homogeneity. We collected cryo-EM data on IBV spike ectodomain, calculated a density map at 3.93Å resolution (Fig 1B; S1 Fig), built an atomic model of the structure and refined it (Fig 1C and 1D). The final structural model contains all of the residues from 21 to 1022 (except residues 702–710) as well as glycans N-linked to 20 sites. Data collection and model statistics are shown in S1 Table.

**Fig 2:**
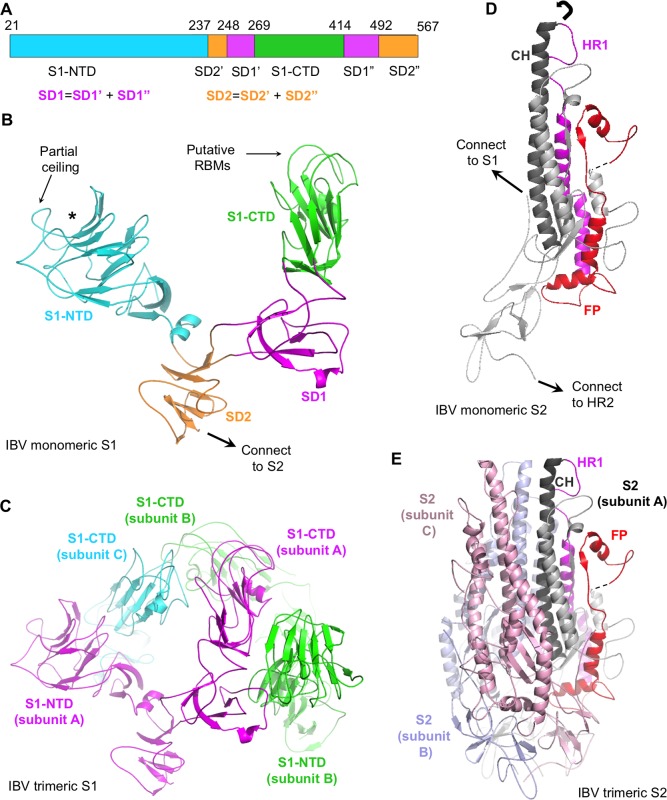
Detailed structure of IBV spike ectodomain. (A) Schematic drawing of IBV S1. SD1: subdomain 1. SD2: subdomain 2. SD1’ and SD1”: two parts of SD1. SD2’ and SD2”: two parts of SD2. (B) Structure of monomeric S1. S1-NTD is colored in cyan. S1-CTD is colored in green. SD1 is colored in magenta. SD2 is colored in orange. * indicates putative sugar-binding site. Partial ceiling on top of the S1-NTD core is labeled. Putative receptor-binding motif loops (RBMs) in S1-CTD are also labeled. (C) Structure of trimeric S1. Three S1 subunits are colored differently. (D) Structure of monomeric S2. The structural elements are colored in the same way as in Fig 1A. (E) Structure of trimeric S2. Dotted line indicates residues 702–710 that are missing in the structural model. The structural elements of subunit A are colored in the same way as in Fig 1D. Subunits B and C are colored in light purple and light pink, respectively. All structures are viewed from the side.

The overall structure of IBV spike ectodomain resembles the pre-fusion structures of coronavirus spikes from the α-, β-, and δ-genera [12–14, 18–20]. It has a clove-like shape, with three S1 heads forming a crown-like structure and sitting on top of a trimeric S2 stalk. Each monomeric subunit of S1 contains two major domains, S1-NTD and S1-CTD, and two subdomains, SD1 and SD2 (Fig 2A and 2B). The S1-CTDs from three different subunits sit on the top and center of the spike trimer, whereas the three S1-NTDs are located on the lower and outer side to S1-CTDs (Fig 2C). SD1 and SD2 connect S1 to S2. The interface of trimeric S2 contains three central helices; each subunit of S2 contains one (Fig 2D and 2E). Each subunit of S2 also contains two heptad repeat regions, HR1 and HR2, and a fusion peptide (FP) (Fig 2D and 2E). In the post-fusion structure of trimeric S2, three copies of HR1 and three copies of HR2 would refold into a six-helix bundle structure, and FP would insert into the target membrane [29–35]. As in the structures of other coronavirus spikes, the HR2 region (residues 1022–1076) in the pre-fusion IBV spike is disordered (Figs 1A and 2D). The exact residue range of coronavirus FP remains unknown, although biochemical studies have identified a region in coronavirus S2 that associates with membranes and likely corresponds to FP (Fig 2D) [48, 49]. In the following sections of this paper, we will compare the structures and functions of IBV spike to those of the spikes from the other three genera, and discuss the evolution of coronavirus spikes.

### Structural and functional evolution of coronavirus spike S1-NTDs

**Fig 3:**
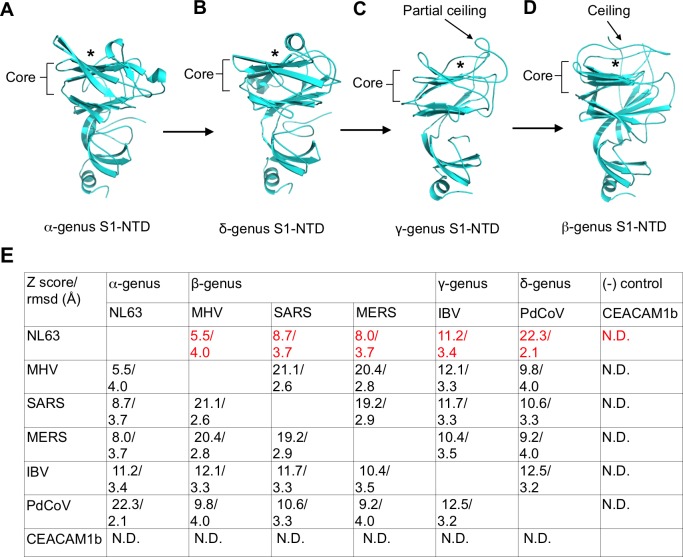
Structural comparisons of S1-NTDs from four coronavirus genera. (A) Structure of S1-NTD from α-genus human coronavirus NL63 (PDB ID: 5SZS). Although each subunit of NL63 S1 contains two copies of S1-NTDs (i.e., S1-NTD1 and S1-NTD2), S1-NTD2 was used in structural comparisons with the S1-NTDs from the other genera because it occupies the same location as the S1-NTDs from the other genera in quaternary structures of the spikes (see Fig 5A). (B) Structure of S1-NTD from δ-genus porcine delta coronavirus (PdCoV) (PDB ID: 6B7N). (C) Structure of S1-NTD from γ-genus IBV. (D) Structure of S1-NTD from β-genus SARS coronavirus (PDB ID: 5X58). * indicates sugar-binding site or putative sugar-binding site in sugar-binding S1-NTDs from each genus. Core structure, partial ceiling, and extensive ceiling are labeled. Arrows from panels (A) to (D) indicate evolutionary direction. (E) Quantitative structural comparisons among S1-NTDs from different genera using software Dali [58]. Both Z-score and r.m.s.d. were calculated for each pair of the proteins. PDB IDs for NL63, PdCoV and SARS S1-NTDs are the same as in panels (A)-(D). PDB IDs for mouse hepatitis coronavirus (MHV) and MERS coronavirus are 3JCL and 5X5F, respectively. CEACAM1b (PDB ID: 5VST), whose β-sandwich fold is topologically different from that of coronavirus S1-NTDs [59], was used as a negative control. N.D.: no detectable structural similarity.

IBV S1-NTD takes the same galectin fold as the S1-NTDs from the other three coronavirus genera, but it contains unique structural features (Fig 3A–3D). Its core structure is a twelve-stranded β-sandwich, which consists of two six-stranded antiparallel β-sheet layers stacked together through hydrophobic interactions (Figs 2B and 3C). The topology of the β-sandwich core is identical to that of human galectins (S2 Fig). Underneath the core structure is another β-sheet and an α-helix, which are also present in the S1-NTDs from the other three coronavirus genera. Above the core structure are some loops that form a partial ceiling-like structure. This structure is not present in human galectins or S1-NTDs from α- or δ-genus, but becomes a more extensive ceiling-like structure in β-coronavirus S1-NTDs (Fig 3A, 3B and 3D). Based on the structure and function of β-coronavirus S1-NTDs, we previously predicted that S1-NTDs from all of the genera have a galectin fold, and further correlated the galectin fold to their functions as viral lectins [15]. Recent structural studies, including the current one, have confirmed our previous structural predictions (S2 Fig). These studies also have unexpectedly revealed that the presence and extent of the ceiling-like structure on top of the core structure are unique structural features for S1-NTDs from different genera.

It has been known that IBV spike binds sugar [50]. A recent study further confirmed that the sugar-binding domain in IBV spike is its S1-NTD [51]. To date no structural information is available for the complexes of coronavirus S1-NTDs and their sugar ligand. Mutagenesis study showed that in the S1-NTD from β-genus bovine coronavirus (BCoV), the sugar-binding site is located in the pocket formed between the core structure and the ceiling [24]. In the structure of host galectins, despite no ceiling, the sugar-binding site is located in the same place [52]. Based on the structural similarity between the S1-NTDs from different coronavirus genera, the sugar-binding site in IBV S1-NTD might also be located in the pocket formed between the core structure and the partial ceiling (Figs 2B and 3C).

The structure determination of IBV S1-NTD provides insight into the structural and functional evolution of coronavirus S1-NTDs. We hypothesized that coronavirus S1-NTDs originated from host galectins based on the structural similarities between coronavirus S1-NTDs and host galectins [23, 24]. As host proteins, galectins are not recognized by the host immune system. In comparison, coronavirus S1-NTDs are under the host immune pressure to evolve. The gradual structural evolution of the ceiling on top of the core structure may have led to three functional outcomes. First, the ceiling could provide better protection to the sugar-binding site from host immune surveillance, which appears to be a common feature of viral lectins [53]. This hypothesis on protected sugar-binding sites in viral lectins is also consistent with the “canyon hypothesis” which states that receptor-binding sites on viral surfaces are hidden from the host immune surveillance [54]. Second, the ceiling is also involved in the quaternary packing of S1, which will be discussed later in this paper. Third, in the structure of S1-NTD from β-genus mouse hepatitis coronavirus (MHV), the outer surface of the ceiling has further evolved the capability to bind a protein receptor CEACAM1, while the presumed sugar-binding pocket has lost its capability to bind sugar [23]. Hence, the structural development of the ceiling is a possible indicator for the evolution of S1-NTDs in the direction of α- and δ-genera, then the γ-genus, and finally the β-genus. Furthermore, we performed quantitative structural comparisons of S1-NTDs from different genera by calculating the Z-score and r.m.s.d. between each pair of the proteins (Fig 3E). The result confirmed that S1-NTDs are relatively conserved among different genera, as reflected by the generally high Z-scores and low r.m.s.d. In terms of structural distances to α-coronavirus S1-NTDs, δ-coronavirus S1-NTDs are the closest, β-coronavirus S1-NTDs are the farthest, and γ-coronavirus S1-NTDs fall in the middle. Moreover, the structural similarity between α- and δ-coronavirus S1-NTDs is slightly higher than that between two β-coronavirus S1-NTDs, suggesting that S1-NTDs within β-genus have diverged slightly more than those between the α- and δ-genera. Taken together, S1-NTDs from the four genera form an evolutionary spectrum in the order of α-, δ-, γ-, and β-genus, with α-coronavirus S1-NTDs probably being the most ancestral (Fig 3A–3D).

### Structural and functional evolution of coronavirus spike S1-CTDs

**Fig 4:**
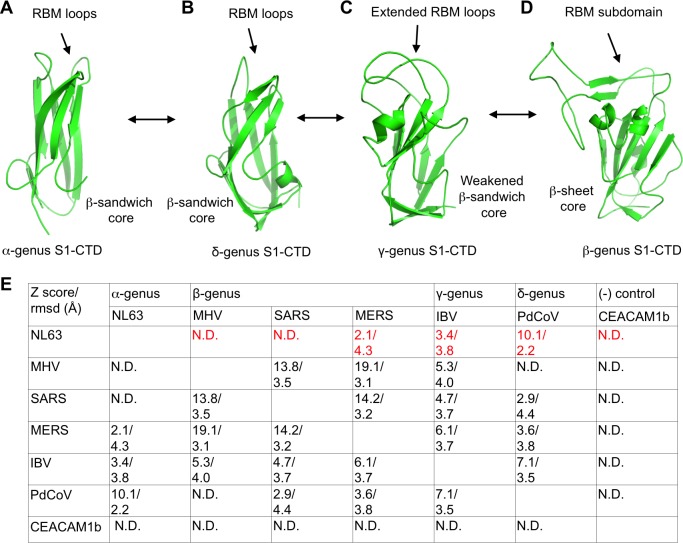
Structural comparisons of S1-CTDs from four coronavirus genera. (A)-(D): Structures of S1-CTDs from different genera. Core structures and RBMs are labeled. (E) Quantitative structural comparisons among S1-CTDs from different genera. The PDB IDs are the same as those in Fig 3. Left right arrows from panels (A) to (D) indicate that evolution could go either way.

The structure of IBV S1-CTD is significantly different from the structures of S1-CTDs from the other genera (Fig 4A–4D; S3 Fig). Its core structure is a β-sandwich containing two β-sheet layers: one is five-stranded and antiparallel, and the other is two-stranded and parallel (Figs 2B and 4C; S3 Fig). The interactions between the two β-sheet layers are present but minimal. In contrast, the core structures of α-coronavirus and δ-coronavirus S1-NTDs are both standard β-sandwich folds with extensive interactions between the two β-sheet layers: one is three-stranded and antiparallel, and the other is three-stranded and mixed (Fig 4A and 4B; S3 Fig). Even more drastically different are the β-coronavirus S1-CTDs, which contain only one five-stranded antiparallel β-sheet layer with the other layer turning into an α-helix and a coil (Fig 4D; S3 Fig). Despite these dramatic structural differences, the S1-CTDs from all genera share the same structural topology (i.e., connectivity of secondary structural elements) (S3 Fig). Moreover, the additional structural motifs on the edge of the core structure are also diverse among different genera (S3 Fig). In the IBV S1-CTD, two extended loops on the edge of the core structure function as putative receptor-binding motifs (RBMs) by potentially binding to an unknown receptor (see below) (Figs 2B and 4C). In both the α- and δ-coronavirus S1-CTDs, three short discontinuous loops are located in the same spatial region; they function as the RBMs in α-coronavirus S1-CTDs and putative RBMs in δ-coronavirus S1-CTDs (Fig 4A and 4B). In β-coronavirus S1-CTDs, a long continuous subdomain is located in this spatial region and functions as the lone RBM (Fig 4D). Structural variations of the RBMs in the S1-CTDs within each of the genera further lead to different receptor specificities [7]. In sum, IBV S1-CTD contains a weakened β-sandwich core structure and two extended RBM loops; the former structural feature falls between the β-sandwich cores of α- and δ-genera and the β-sheet core of β-genus, whereas the latter structural feature falls between the three short discontinuous RBM loops of α- and δ-genera and a single long continuous RBM subdomain of β-genus.

To investigate the function of IBV S1-CTD, we performed two assays to detect possible interactions between IBV S1-CTD and its potential receptor on the host cell surface. First, we carried out an IBV-spike-mediated pseudovirus entry assay in the presence of recombinant IBV S1–CTD (S4A Fig). To this end, retroviruses pseudotyped with IBV spike (i.e., IBV pseudoviruses) were used to enter host cells. In the absence of recombinant IBV S1-CTD, IBV pseudoviruses entered DF-1 cells (chicken fibroblast) efficiently, which was consistent with a previous report showing that DF-1 cells are permissive to live IBV (strain M41) infections [55]. As a negative control, their entry into HEK293T cells (human kidney) was inefficient. Recombinant IBV S1-CTD reduced the efficiency of IBV pseudovirus entry into DF-1 cells in a dose-dependent manner, likely because it competed with IBV pseudoviruses for an unknown receptor on the host cell surface. Second, we examined the binding of recombinant IBV S1-CTD to the host cell surface using a flow cytometry assay (S4B Fig). To this end, recombinant IBV S1-CTD was incubated with DF-1 cells, and subsequently cell-bound S1-CTD was detected using flow cytometry. Recombinant IBV S1-CTD bound to the surface of DF-1 cells efficiently, but not the surface of HEK293T cells. Taken together, IBV S1-CTD binds to a yet-to-be-identified receptor on the surface of chicken cells and hence functions as a receptor-binding domain (RBD). Thus, the S1-NTD and S1-CTD of IBV spike may both function as RBDs. Because coronavirus S1-CTDs from the α- and β-genera all use the additional structural features on the edge of their core structure as their RBMs, it is likely that the two extended loops in the same spatial region in IBV S1-CTD function as the RBMs.

Coronavirus S1-CTDs represent remarkable examples of divergent evolution of viral proteins. The core structures and the RBM regions of S1-CTDs are both divergent among different genera (Fig 4A–4D; S3 Fig). The core structures are β-sandwiches for α- and δ-coronavirus S1-CTDs, weakened β-sandwiches for γ-coronavirus S1-CTDs, and single β-sheet layer for β-coronavirus S1-CTDs. The RBMs are three short discontinuous loops for α- and δ-coronavirus S1-CTDs, two reinforced loops for γ-coronavirus S1-CTDs, and a single continuous subdomain for β-coronavirus S1-CTDs. Hence the S1-CTDs form an evolutionary spectrum, with α- and δ-coronavirus S1-CTDs on one end, β-coronavirus S1-CTDs on the other end, and γ-coronavirus S1-CTDs in between. We performed quantitative structural comparisons of S1-CTDs from all four genera (Fig 4E). The result confirmed that S1-CTDs are relatively poorly conserved among different genera, as reflected by the generally low Z-scores and high r.m.s.d. In terms of structural distances to α-coronavirus S1-CTDs, δ-coronavirus S1-CTDs are the closest, β-coronavirus S1-CTDs are the farthest, and γ-coronavirus S1-CTDs fall in the middle. The functional outcomes of the core structure evolution are not clear, but the evolution of the RBMs may have led to the following two functional outcomes. First, the diversity of the RBMs from three short loops to two extended loops and then to a long subdomain may allow coronaviruses to explore a wider variety of receptors. Second, the reinforced RBM regions in both β- and γ-coronavirus S1-NTDs facilitate quaternary packing of S1, which will be discussed later in this paper. Taken together, the S1-CTDs from different genera form an evolutionary spectrum in the order of α-, δ-, γ-, and β-genus, although the evolutionary direction could go either way (Fig 4A–4D).

### Evolution of quaternary packing of coronavirus S1

**Fig 5:**
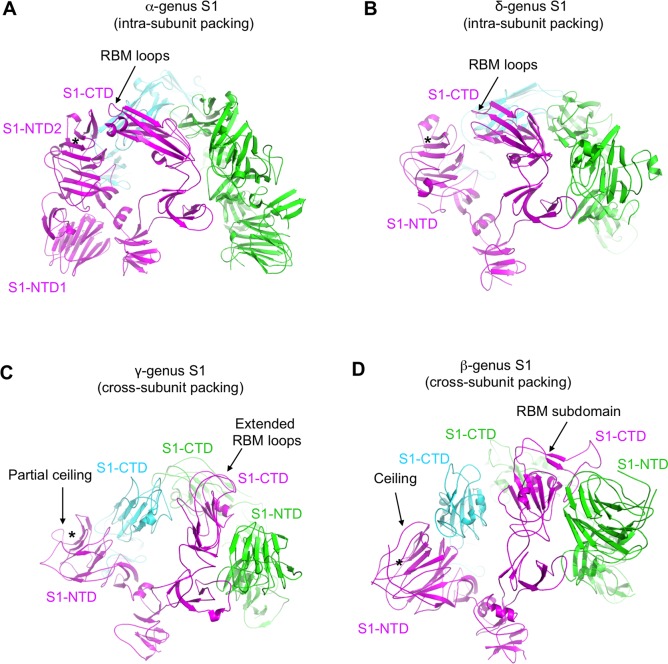
Quaternary packing of S1 from four coronavirus genera. (A)-(D) Structures of trimeric S1 from different genera. Three S1 subunits are colored differently. The PDB IDs are the same as those in Fig 3. All structures are viewed from the side.

Curiously, coronavirus S1 from different genera take two types of quaternary packing modes (Fig 5A–5D) [12–14, 18–20]. IBV S1 takes a cross-subunit quaternary packing mode where the S1-NTD and S1-CTD from different subunits pack together (Fig 5C). Specifically, in the trimeric IBV spike, one S1-CTD packs against two S1-CTDs from the other subunits as well as one S1-NTD from another subunit. The putative RBMs of IBV S1-CTD and the partial ceiling of IBV S1-NTD are both involved in the cross-subunit packing. As a result, the putative RBMs of IBV S1-CTD are partially concealed, disallowing their full access to the host receptor. Hence IBV S1-CTD in the current structure was captured in a “lying down” state, and would need to “stand up” on the spike trimer for efficient receptor binding. This potential conformational change of IBV S1 can minimize the exposure of the putative RBMs in its S1-CTD to the immune system, thereby functioning as a possible strategy for viral immune evasion. β-coronavirus S1 also takes the cross-subunit packing mode, with the RBM of its S1-CTD and the ceiling of its S1-NTD both involved in the cross-subunit packing (Fig 5D) [18–20]. In contrast, α- and δ-coronavirus S1 both take an intra-subunit packing mode where the S1-NTD and S1-CTD from the same subunit pack together (Fig 5A and 5B) [12–14]. The RBMs of α- and δ-coronavirus S1-CTDs are involved in the intra-subunit packing. Whether S1 packs in the intra-subunit or cross-subunit mode, the RBMs of S1-CTDs are concealed or partially concealed in their “lying down” state, and would need to switch to the “standing up” state for receptor binding. Overall, β- and γ-coronavirus S1 both take the cross-subunit quaternary packing mode, whereas α- and δ-coronavirus S1 both take the intra-subunit quaternary packing mode.

We examined whether the quaternary structures of coronavirus S1 can lead to functional differences of coronavirus spikes. First, in both β- and γ-coronavirus spikes, the RBMs of their S1-CTDs and the ceiling of their S1-NTDs have evolved to facilitate the cross-subunit packing. These additional structural features further evolved to gain other functions: the RBMs of S1-CTDs recognize diverse protein receptors, whereas the ceiling of the S1-NTDs either protects the sugar-binding site or recognizes a new protein receptor [7]. Second, to investigate the structural restrain on S1-CTDs that may hinder their potential conformational change, we calculated the total and buried surface areas of the S1-CTD on the spikes from different genera. The result did not reveal systematic difference between intra-subunit S1 packing and cross-subunit S1 packing in the buried surface area of S1-CTDs. However, it is worth noting that the S1-CTD from β-genus SARS-CoV has the smallest buried surface area (in both absolute value and percentage) (S2 Table). The relative small buried surface area of SARS-CoV S1-CTD indicates less structural restraint on the S1-CTD from other parts of the spike S1, possibly allowing the S1-CTD to switch to the “standing up” and receptor-accessible conformation more easily. The “standing up” conformation of SARS-CoV S1-CTD may also weaken the structural restraint of S1 on S2 (discussed in more detail later), potentially allowing membrane fusion to proceed more easily [56]. Indeed, frequent “standing up” of SARS-CoV S1-CTD has been observed [19]. Overall, compared to the intra-subunit quaternary packing of α- and δ-coronavirus S1, the cross-subunit quaternary packing of β- and γ-coronavirus S1 may have allowed their S1 to evolve additional functions in receptor recognition; moreover, the S1-CTD from β-genus SARS-CoV spike has a relatively small buried surface area, which may be responsible for its dynamic receptor-binding conformation.

### Structural and functional evolution of coronavirus spike S2

The structure and function of IBV S2 are highly similar to those of S2 from the other coronavirus genera. In the pre-fusion structure of IBV S2, HR2 is disordered, whereas HR1 and FP each consist of several α-helices and connecting loops (the exact residue range of FP is not clear) (Fig 2D). In the post-fusion structure, HR1 would refold into a long α-helix, HR2 would refold into a mixture of α-helices and coils, three copies of HR1 and HR2 would pack into a six-helix bundle structure, and FP would also refold and insert into the target membrane (S5A Fig) [6, 29]. IBV S2 is locked in the pre-fusion state because of the structural restraint from S1. Specifically, because of the cross-subunit quaternary packing of trimeric IBV S1, HR1 and FP of IBV S2 are structurally restrained by two S1-CTDs from the other subunits and SD1 from another subunit, respectively (S5B Fig). The structural restraints from S1 on S2 can be weakened by the standing up of S1-CTDs (which allows receptor binding) and can be lifted completely upon proteolysis removal of S1. The packing between S1 and S2 in IBV spike is the same as those in β-coronavirus spikes [18–20]. However, in α- and δ-coronavirus spikes, the packing between S1 and S2 becomes different due to the intra-subunit quaternary packing of their trimeric S1: HR1 and FP are restrained by one S1-CTD and one SD1 from another subunit, respectively (S5C Fig) [12–14]. Other than the differences in S1/S2 packing, the structural and functional similarities of coronavirus S2 from different genera suggest evolutionary conservation of coronavirus S2.

## Discussion

The fast evolutionary rates of viruses, particularly RNA viruses, make it difficult to trace their evolutionary history [1–3]. Envelope-anchored coronavirus spike proteins guide viral entry into cells; they are the fastest evolving coronavirus proteins due to viral needs to engage diverse host receptors, maximize membrane-fusion efficiency, and evade host immune surveillance [7–14]. Coronavirus spikes from four different genera are divergent, and their evolutionary relationships pose a major puzzle in the virology field [6]. Because viral proteins need to function under certain structural and functional constraints, evolutionary information of viral proteins can be more reliably found in their tertiary structures and related functions than in their primary structures [16, 17]. Although extensive structural studies including both X-ray crystallography and cryo-EM have been done on coronavirus spikes, a critical piece that was still missing is the structure of γ-coronavirus spikes, preventing a clear understanding of the evolutionary relationships among coronavirus spikes [12–14, 18–28]. In this study, we determined the cryo-EM structure of IBV spike ectodomain, the first such structure from the γ-genus, which bridges the divergent structures of coronavirus spikes into an evolutionary spectrum and provides insight into the evolutionary relationships among coronavirus spikes.

Our study compares the structures and functions of coronavirus spikes from the four genera, and illustrates the structural and functional evolution of these proteins. First, coronavirus S1-NTDs from all genera share the same structural fold and possibly evolutionary origins with host galectins. From α- and δ- genera to γ genus and then to β genus, the S1-NTDs have evolved from simple galectin-fold core structure with an exposed sugar-binding site, to having a partial ceiling on top of the core structure, and to having an extensive ceiling to protect the sugar-binding site from host immune surveillance (the outer surface of the ceiling in one β-coronavirus can even bind to a novel protein receptor). The partial ceiling in γ-coronavirus S1-NTDs and the extensive ceiling in β-coronavirus S1-NTDs are also involved in the quaternary packing of S1. Second, coronavirus S1-CTDs from different genera are very diverse, but still form an evolutionary spectrum with α- and β-coronavirus S1-CTDs at two ends and δ- and γ-coronavirus S1-CTDs in the middle. The core structures of S1-CTDs have diverged from β-sandwich to weakened β-sandwich and then to β-sheet, whereas the RBMs have diverged from short loops to extended loops and then to a long subdomain. The functional significance of the core structure evolution is not clear, but the RBM evolution may allow the viruses to expand receptor recognition and also contributes to the quaternary packing of S1. Third, from α- and δ- genera to β- and γ-genera, the quaternary packing of S1 has diverged from simple intra-subunit packing to more complex cross-subunit packing. The cross-subunit quaternary packing of β- and γ-coronavirus S1 may have allowed their S1 to evolve additional functions in receptor recognition. Moreover, the relatively small buried surface area of the S1-CTD from β-genus SARS-CoV may allow the S1-CTD to be more dynamic for receptor binding. Finally, the S2 from all four genera are structurally and functionally conserved, although there are some differences in their S1/S2 packing. Quantitative structural comparisons also demonstrate that in terms of structural distances to α-coronavirus S1, δ-coronavirus S1 is the closest, β-coronavirus S1 is the farthest, and γ-coronavirus S1 is the intermediate. We also calculated the phylogenetic tree using the amino acid sequences of 29 coronavirus spikes from different genera, and the result showed that in terms of amino acid sequence distances to α-coronavirus spikes, δ-coronavirus spike is the closest, β-coronavirus spike is the farthest, and γ-coronavirus spike is the intermediate (S6 Fig). Taken together, coronavirus spikes from different genera form an evolutionary spectrum, with α-coronavirus spikes on one end, followed by δ-coronavirus spikes and γ-coronavirus spikes, and β-coronavirus spikes on the other end.

Because of their fast evolutionary rates, viruses are perfect model systems for studying evolution. Our study has demonstrated that despite structural divergence among coronavirus spikes, particularly in their S1 where low or little structural similarities can be detected, we can still trace the evolutionary relationships among these viral proteins through detailed analyses of their structures and functions. Our study also reveals that coronavirus spikes have evolved to remarkable diversity to expand their receptor recognition, facilitate membrane fusion, and evade host immune surveillance, while conserving basic membrane-fusion mechanisms. The evolutionary approaches that coronaviruses take and the evolutionary edges that they gain are good examples of viral evolution and deepen our understanding of evolution in general.

## Supporting information

S1 Table Data collection and model validation statistics. (DOC)

S2 Table Buried surface area of coronavirus spike S1-CTDs. (DOC)

S1 Fig Cryo-EM data analysis of IBV spike ectodomain. (A) Representative micrograph of frozen-hydrated IBV spike ectodomain particles (top) and representative 2D class averages in different orientations (bottom). (B) Gold-standard Fourier shell correlation (FSC) curves. The resolution was determined to be 3.93 Å. The 0.143 cut-off value is indicated by a horizontal red bar.
(TIF)

S2 Fig Structural topology of coronavirus S1-NTDs. (A) Structural topology of the core structure of human galectin-3 (PDB ID: 1A3K). (B) Structural topology of the core structures of α-, γ-, and δ-coronavirus S1-NTDs. (C) Structural topology of the core structures of β-coronavirus S1-NTD. PDB IDs of coronavirus S1-NTDs are the same as in Fig 3. β-strands are shown as arrows. The two layers of the core structures are colored in green and magenta, respectively. N* and C* indicate N- and C-terminus, respectively. Numbering of the secondary structures only counts secondary structural elements in the core region.
(TIF)

S3 Fig Structural topology of coronavirus S1-CTDs. (A) Structural topology of the core structures of α- and δ-coronavirus S1-CTDs. (B) Structural topology of the core structure of γ-coronavirus S1-CTD. (C) Structural topology of the core structure of β-coronavirus S1-CTD. PDB IDs of coronavirus S1-CTDs are the same as in Fig 4. β-strands are shown as arrows. α-helices are shown as cylinders. Coil is shown as a curled line. The two layers of the core structures are colored in green and magenta, respectively. Receptor-binding motifs (RBMs) are colored in red and the relative lengths of the RBMs are labeled in parentheses. In both γ- and δ-coronavirus S1-CTDs, the RBMs have not been experimentally identified and thus their functions are putative. N* and C* indicate N- and C-terminus, respectively. Numbering of the secondary structures only counts secondary structural elements in the core region.
(TIF)

S4 Fig Function of IBV S1-CTD. (A) IBV pseudovirus entry into cells in the presence of recombinant IBV S1-CTD. Entry efficiency was characterized by luciferase activity accompanying entry. RLU: relative light units. Mock: no IBV pseudoviruses were added. Entry: IBV pseudovirus entry in the absence of any recombinant IBV S1-CTD. (B) Flow cytometry assay for the binding of recombinant IBV S1-CTD to the surface of cells. Cell-bound IBV S1-CTD was detected using antibodies recognizing its C-terminal His_6_ tag. Cells only or cells plus antibody without IBV S1-CTD were used as negative controls. Statistic analyses were performed using two-tailed t-test. Error bars indicate S.E.M. (n = 4). *** *P*<0.001. ** *P*<0.01. * *P*<0.05. N.S.: no statistical significance.
(TIF)

S5 Fig Structure and function of IBV S2. (A) Structures of monomeric β-genus MHV S2 in the pre-fusion conformation (left; PDB ID: 3JCL) and post-fusion conformation (right; PDB ID: 6B3O). Structural elements in monomeric S2 are colored in the same way as in Fig 2D. Arrow in the pre-fusion structure indicates the direction in which HR1 would need to extend to reach the post-fusion conformation. (B) Packing between S1 and S2 in IBV spike. Trimeric S1 and one monomeric S2 are shown. Structural elements in monomeric S2 are colored in the same way as in panel (A). Three S1 subunits are colored differently. (C) Packing between S1 and S2 in porcine delta coronavirus spike (PDB ID: 6B7N). Trimeric S1 and one monomeric S2 are shown. S1 and S2 are colored in the same way as in panel (B). All structures are viewed from the side.
(TIF)

S6 Fig Phylogenetic tree derived from the amino acid sequences of 29 coronavirus spikes. The phylogenetic tree was constructed using the neighbor-joining method as previously described [57]. Horizontal scale bars represent average numbers of substitutions per amino acid position. The GenBank accession numbers of the selected spikes are marked before each virus name.
(TIF)
