# Supplementary material for: Cryo-EM structure of infectious bronchitis coronavirus spike protein reveals structural and functional evolution of coronavirus spike proteins
Source: PLoS Pathog. 2018 Apr 23;14(4):e1007009. doi: 10.1371/journal.ppat.1007009 (PMC5933801; doi:10.1371/journal.ppat.1007009)
Supplement: S1 Table — (DOC) [file ppat.1007009.s001.doc]

**Table S1. Data collection and model validation statistics**

| **Data Collection** |  | |
| --- | --- | --- |
| Microscope | Titan Krios | |
| Voltage (keV) | 300 | |
| Camera | Gatan K2 | |
| Camera model | Super-resolution | |
| Defocus range (µm) | 1.0 to 4.0 | |
| Exposure time (s) | 10 | |
| Movies | 2,037 | |
| Frames per movie | 50 | |
| Dose rate (*e*^-^/Å^2^/s) | 5.366 | |
| Total dose per movie (*e*^-^/Å^2^) | 53.66 | |
| Pixel size (Å) | 0.68 | |
| **Reconstruction** |  | |
| Software | RELION 2.1 | |
| Symmetry | C3 | |
| Particles refined | 102,471 | |
| Map Resolution (Å) | 3.93 | |
| Map sharpening *B*-factor (Å^2^) | | -205.316 |
| **Model Validation** |  | |
| UCSF Chimera CC | 0.8077 | |
| EMRinger Score | 3.09 | |
| MolProbity Score | 1.84 | |
| All-atom clashscore | 4.28 | |
| C_β_ deviations | 0 | |
| Rotamer outliers (%) | 0.51 | |
| Ramachandran |  | |
| Favored (%) | 86.55 | |
| Allowed (%) | 13.25 | |
| Outliers (%) | 0.20 | |
| RMS deviations |  | |
| Bond length (Å) | 1.355 | |
| Bond angles (°) | 0.006 | |
