# Supplementary material for: Cryo-EM structure of infectious bronchitis coronavirus spike protein reveals structural and functional evolution of coronavirus spike proteins
Source: PLoS Pathog. 2018 Apr 23;14(4):e1007009. doi: 10.1371/journal.ppat.1007009 (PMC5933801; doi:10.1371/journal.ppat.1007009)
Supplement: S2 Table — (DOC) [file ppat.1007009.s002.doc]

**Table S2. Buried surface area of coronavirus spike S1-CTDs**

| S1-CTD | α-genus | β-genus | | γ-genus | δ-genus |
| --- | --- | --- | --- | --- | --- |
|  | NL63 | SARS-CoV | MHV | IBV | PdCoV |
| Total surface area (Å^2^) | 7074.7 | 10792.7 | 15337.3 | 7993.0 | 6498.3 |
| Buried surface area (Å^2^) | 881.1 | **806.4** | 1259.6 | 1166.8 | **1284.1** |
| Percentage of buried surface area (%) | 12.5 | **7.5** | 8.2 | 13.3 | **19.8** |
