# Supplementary figures and images for: Cryo-EM structure of infectious bronchitis coronavirus spike protein reveals structural and functional evolution of coronavirus spike proteins
Source: PLoS Pathog. 2018 Apr 23;14(4):e1007009. doi: 10.1371/journal.ppat.1007009 (PMC5933801; doi:10.1371/journal.ppat.1007009)

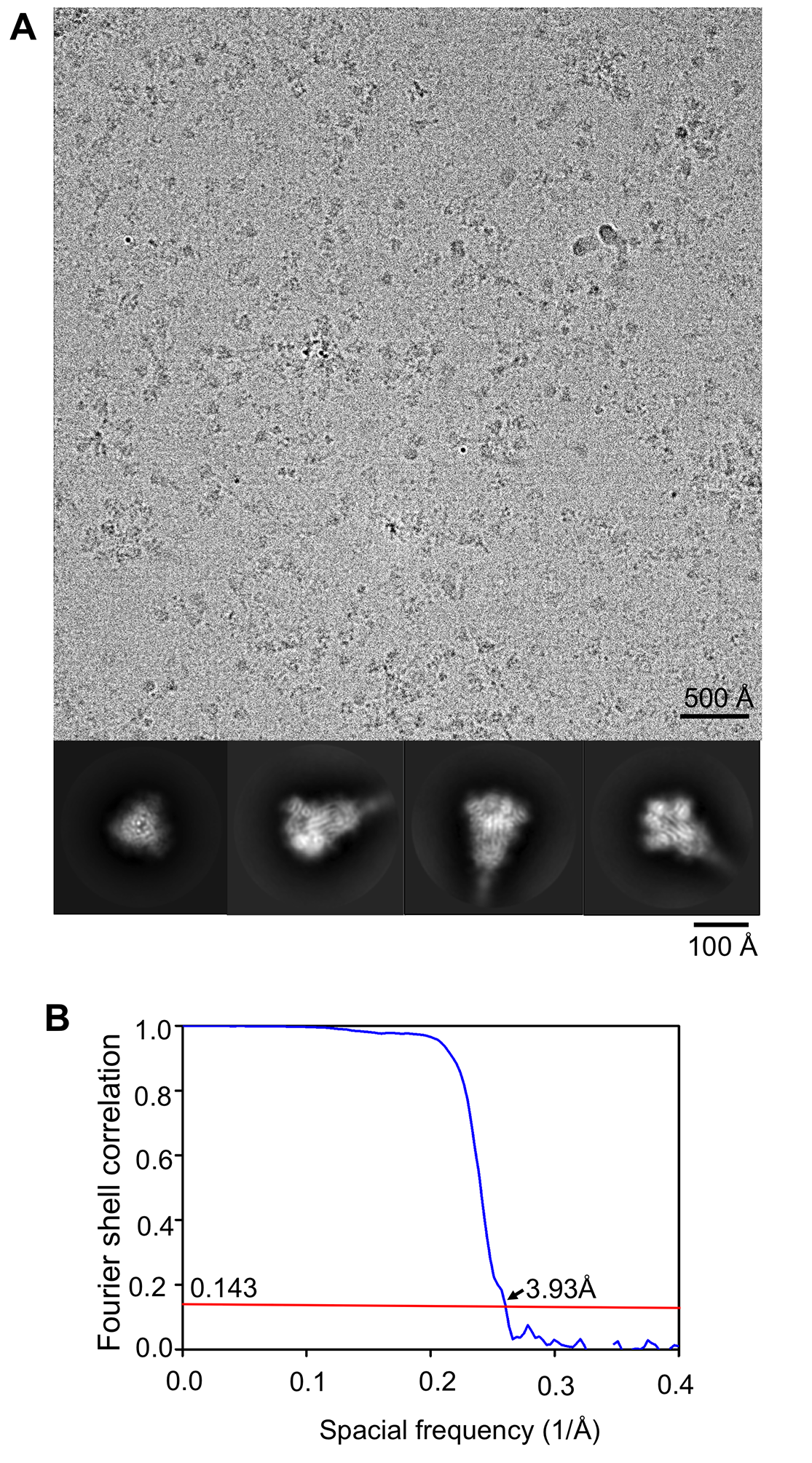

Supplement: S1 Fig — (A) Representative micrograph of frozen-hydrated IBV spike ectodomain particles (top) and representative 2D class averages in different orientations (bottom). (B) Gold-standard Fourier shell correlation (FSC) curves. The resolution was determined to be 3.93 Å. The 0.143 cut-off value is indicated by a horizontal red bar. (TIF) [file ppat.1007009.s003.tif]

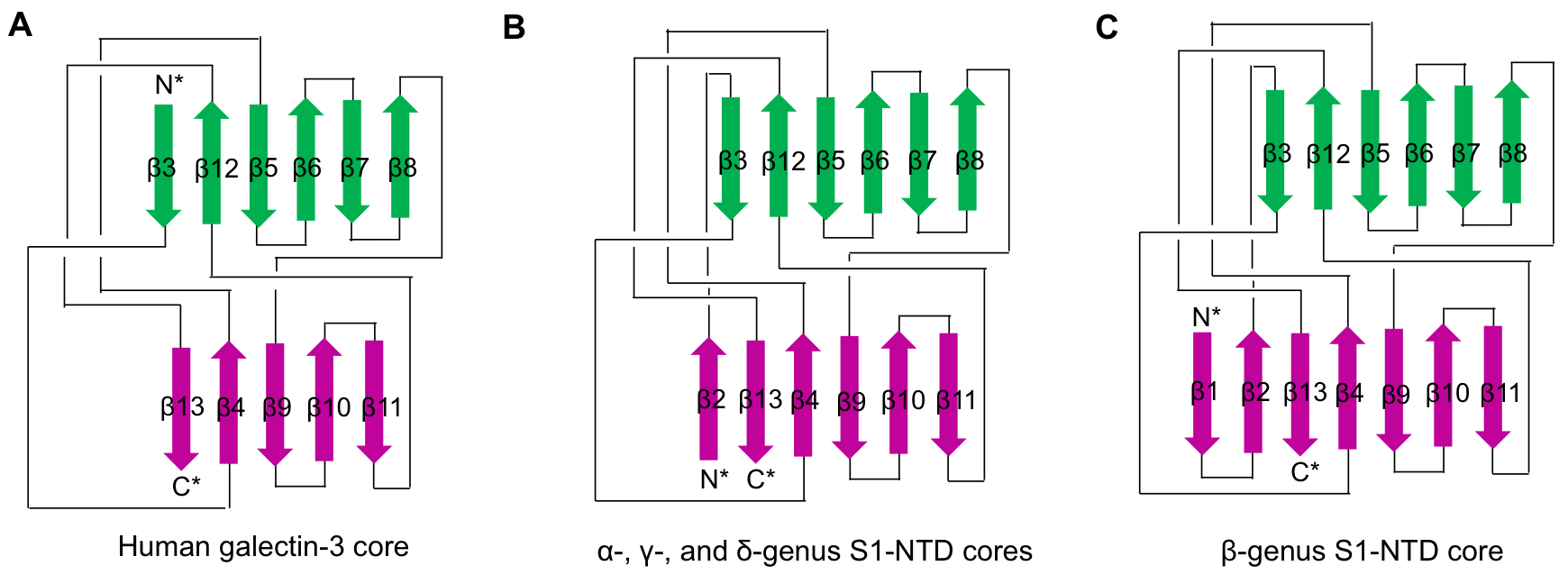

Supplement: S2 Fig — (A) Structural topology of the core structure of human galectin-3 (PDB ID: 1A3K). (B) Structural topology of the core structures of α-, γ-, and δ-coronavirus S1-NTDs. (C) Structural topology of the core structures of β-coronavirus S1-NTD. PDB IDs of coronavirus S1-NTDs are the same as in Fig 3. β-strands are shown as arrows. The two layers of the core structures are colored in green and magenta, respectively. N* and C* indicate N- and C-terminus, respectively. Numbering of the secondary structures only counts secondary structural elements in the core region. (TIF) [file ppat.1007009.s004.tif]

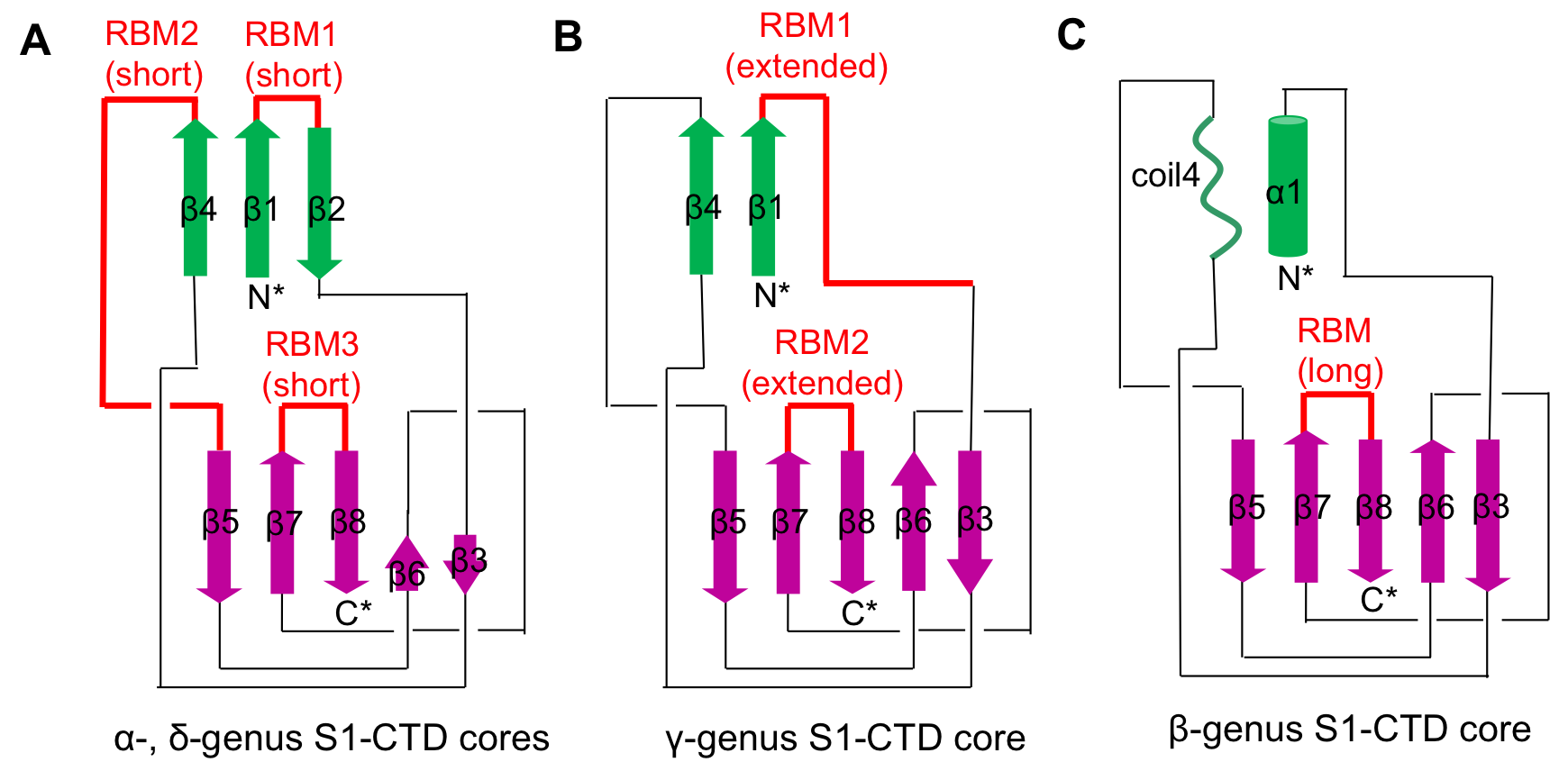

Supplement: S3 Fig — (A) Structural topology of the core structures of α- and δ-coronavirus S1-CTDs. (B) Structural topology of the core structure of γ-coronavirus S1-CTD. (C) Structural topology of the core structure of β-coronavirus S1-CTD. PDB IDs of coronavirus S1-CTDs are the same as in Fig 4. β-strands are shown as arrows. α-helices are shown as cylinders. Coil is shown as a curled line. The two layers of the core structures are colored in green and magenta, respectively. Receptor-binding motifs (RBMs) are colored in red and the relative lengths of the RBMs are labeled in parentheses. In both γ- and δ-coronavirus S1-CTDs, the RBMs have not been experimentally identified and thus their functions are putative. N* and C* indicate N- and C-terminus, respectively. Numbering of the secondary structures only counts secondary structural elements in the core region. (TIF) [file ppat.1007009.s005.tif]

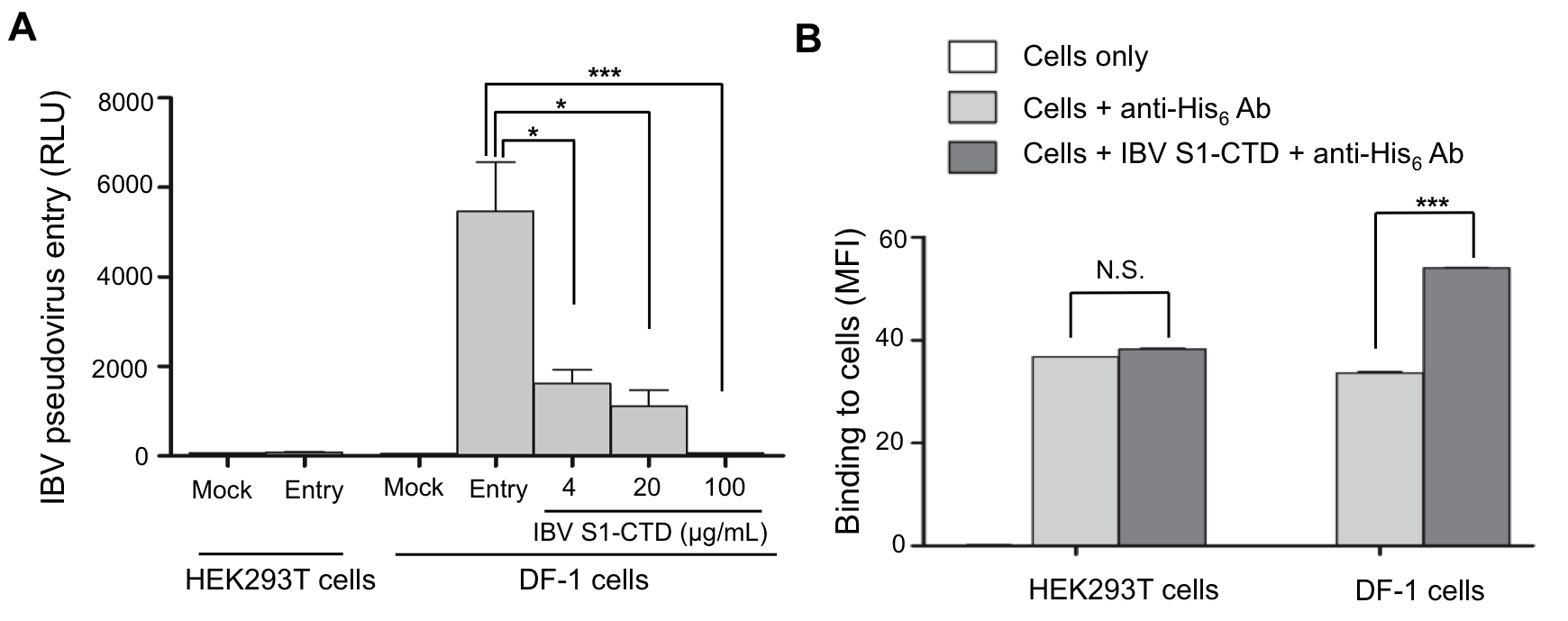

Supplement: S4 Fig — (A) IBV pseudovirus entry into cells in the presence of recombinant IBV S1-CTD. Entry efficiency was characterized by luciferase activity accompanying entry. RLU: relative light units. Mock: no IBV pseudoviruses were added. Entry: IBV pseudovirus entry in the absence of any recombinant IBV S1-CTD. (B) Flow cytometry assay for the binding of recombinant IBV S1-CTD to the surface of cells. Cell-bound IBV S1-CTD was detected using antibodies recognizing its C-terminal His6 tag. Cells only or cells plus antibody without IBV S1-CTD were used as negative controls. Statistic analyses were performed using two-tailed t-test. Error bars indicate S.E.M. (n = 4). *** P<0.001. ** P<0.01. * P<0.05. N.S.: no statistical significance. (TIF) [file ppat.1007009.s006.tif]

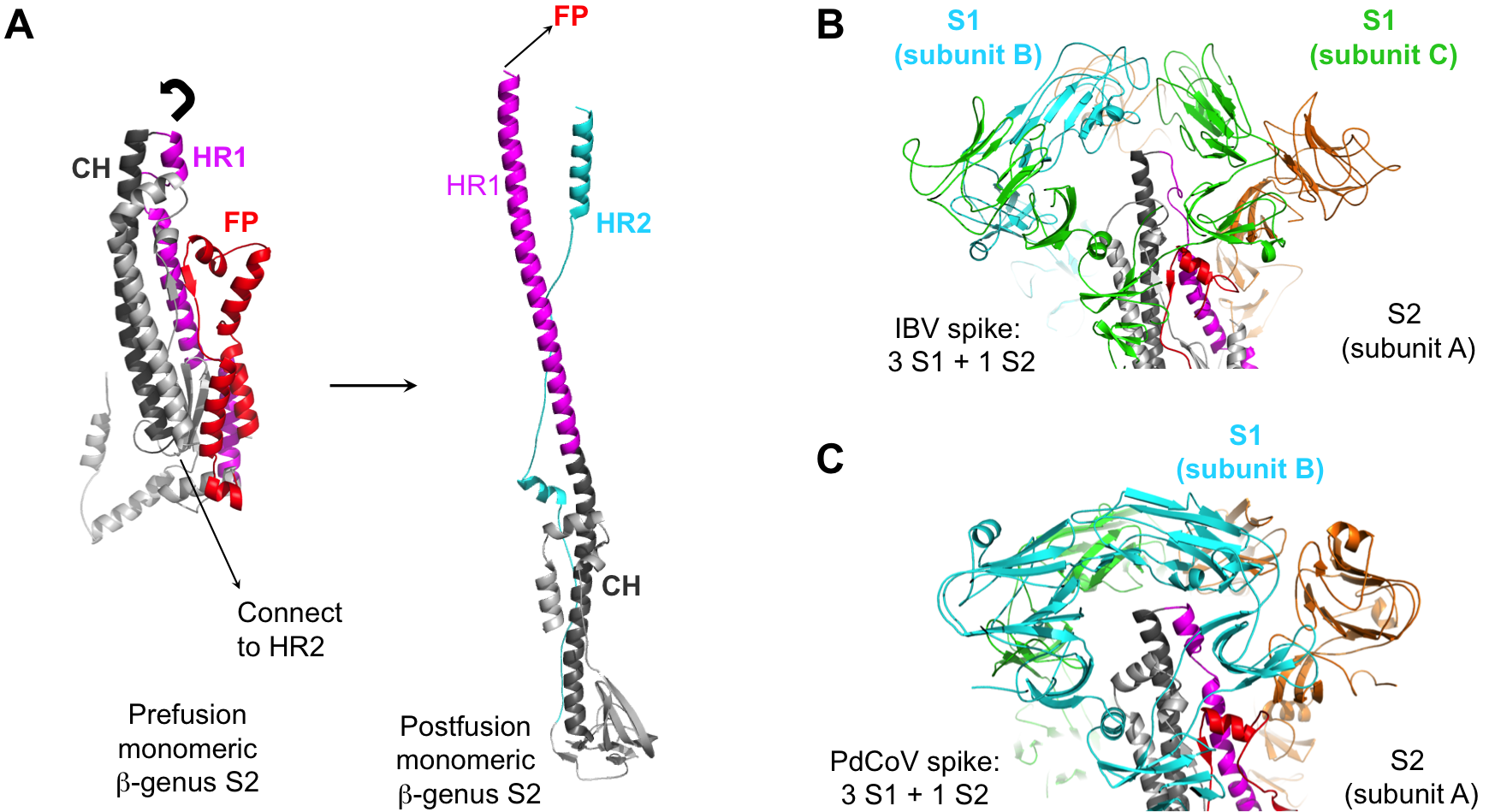

Supplement: S5 Fig — (A) Structures of monomeric β-genus MHV S2 in the pre-fusion conformation (left; PDB ID: 3JCL) and post-fusion conformation (right; PDB ID: 6B3O). Structural elements in monomeric S2 are colored in the same way as in Fig 2D. Arrow in the pre-fusion structure indicates the direction in which HR1 would need to extend to reach the post-fusion conformation. (B) Packing between S1 and S2 in IBV spike. Trimeric S1 and one monomeric S2 are shown. Structural elements in monomeric S2 are colored in the same way as in panel (A). Three S1 subunits are colored differently. (C) Packing between S1 and S2 in porcine delta coronavirus spike (PDB ID: 6B7N). Trimeric S1 and one monomeric S2 are shown. S1 and S2 are colored in the same way as in panel (B). All structures are viewed from the side. (TIF) [file ppat.1007009.s007.tif]

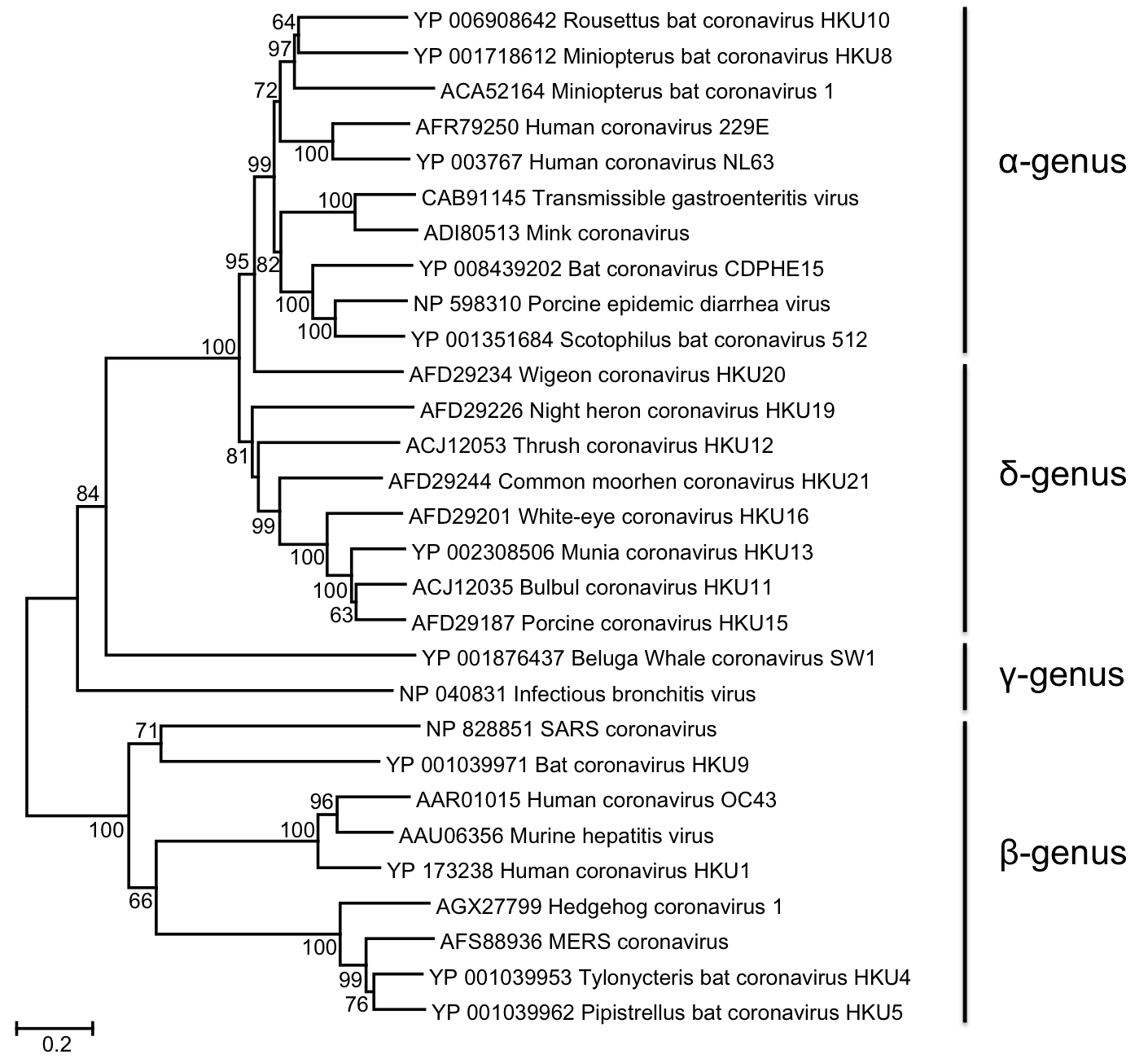

Supplement: S6 Fig — The phylogenetic tree was constructed using the neighbor-joining method as previously described [57]. Horizontal scale bars represent average numbers of substitutions per amino acid position. The GenBank accession numbers of the selected spikes are marked before each virus name. (TIF) [file ppat.1007009.s008.tif]
